# Effects of Common Food Additives Kappa‐, Iota‐ and Lambda‐Carrageenans on Intestinal Epithelial Cell Activation and Barrier Disruption

**DOI:** 10.1111/cea.70266

**Published:** 2026-04-06

**Authors:** Na Sun, Yagiz Pat, Huseyn Babayev, Sena Ardicli, Ismail Ogulur, Xianting Bu, Manru Li, Shuqi Jia, Beate Rückert, Raja Dhir, Kari C. Nadeau, Mubeccel Akdis, Cezmi A. Akdis, Duygu Yazici

**Affiliations:** ^1^ Swiss Institute of Allergy and Asthma Research (SIAF) University of Zurich Davos Switzerland; ^2^ SKL of Marine Food Processing & Safety Control, National Engineering Research Center of Seafood, School of Food Science and Technology Dalian Polytechnic University Dalian P. R. China; ^3^ Department of Genetics, Faculty of Veterinary Medicine Bursa Uludag University Bursa Turkey; ^4^ Department of Environmental Health, T.H. Chan School of Public Health Harvard University Boston Massachusetts USA

**Keywords:** carrageenan, epithelial barrier, food emulsifiers, gut‐on‐a‐chip, inflammation, RNA‐sequencing, targeted proteomics

## Abstract

**Background:**

The epithelial barrier theory has been proposed to link the onset of many chronic inflammatory diseases to the damaged epithelial barrier induced by numerous environmental factors, including food emulsifiers. This study aimed to investigate the effects of food emulsifiers kappa‐, iota‐ and lambda‐carrageenan on intestinal epithelial barrier integrity and the underlying mechanisms.

**Methods:**

The cytotoxicity and apoptosis of carrageenans were determined using Hoechst/propidium iodide staining and FITC annexin V/propidium iodide staining, respectively. The gut‐on‐a‐chip was established and transepithelial electrical resistance measurement, paracellular flux assay, immunofluorescence staining of tight junction proteins, RNA‐sequencing and targeted proteomics were performed.

**Results:**

Kappa‐, iota‐ and lambda‐carrageenans induced dose‐dependent cytotoxicity, with lambda‐carrageenan showing a more severe cytotoxic effect in relatively lower doses. All three carrageenans elicited a dose‐ and time‐dependent decrease of gut epithelial barrier strength, a significant increase in the paracellular flux and irregular and heterogeneous staining of occludin and ZO‐1 compared to the untreated group, suggesting the disruption of the intestinal epithelial barrier integrity. Transcriptomics data revealed that iota‐ and lambda‐carrageenan induced more severe pro‐inflammatory responses, which were associated with the upregulation of genes involved in TNF signalling, IL‐17 signalling cellular response to chemokines, cholesterol metabolism and NF‐kappa B signalling pathways. Targeted proteomics data from exposed gut‐on‐a‐chips indicated an upregulation of inflammation‐ and immune response‐related proteins for all three carrageenans.

**Conclusions:**

The present study provides direct evidence for the detrimental effects of kappa‐, iota‐ and lambda‐carrageenans on gut epithelial cell activation and barrier integrity. The underlying mechanism of epithelial barrier disruption was largely attributed to the activation of innate immune responses by carrageenans, resulting in a pro‐inflammatory response.

## Key Messages

- Carrageenans show dose‐dependent cytotoxicity in intestinal epithelial cells; lambda‐carrageenan is the most potent.
- Carrageenans disrupt intestinal barrier integrity and increase paracellular permeability.
- Carrageenans activate innate immune signalling and pro‐inflammatory responses; kappa carrageenan has a better cellular toxicity and innate immune response inducing profile.

## Graphical abstract

Carrageenans, widely used food additives, disrupted intestinal epithelial integrity in a gut‐on‐a‐chip model. All types (κ‐, ɩ‐, λ‐) induced cytotoxicity, inflammation and tight junction (TJ) disruption, triggering TNF‐mediated immune responses. λ‐Carrageenan had the most severe effects, supporting the Epithelial Barrier Theory linking food additives to chronic inflammatory diseases.

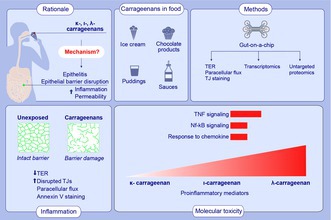

## 1 Introduction

Many chronic non‐communicable diseases have shown a steady rise in their prevalence since the 1960s, currently affecting over 25% of the world's population [1, 2]. Since the widespread industrialization of chemical manufacturing in the 1950s, over 350,000 new substances have been registered for production and use, and encountered by humans without any major health concerns [3]. The recently proposed ‘Epithelial Barrier Theory’ gives a scientific explanation for the growing prevalence and major health care burden of many chronic non‐communicable diseases, attributing the onset and exacerbations of these diseases to the impaired epithelial barrier [1, 4, 5]. These diseases are triggered by exposure to certain toxic substances in the environment, such as air pollution, cigarette smoke, volatile organic compounds, ozone, microplastics, nanoplastics, detergents, surfactants, enzymes, household cleaners, hand sanitizers, disinfectants and food additives in processed foods such as emulsifiers, all introduced by industrialization, urbanisation, and westernised lifestyles [6].

Processed foods account for a high proportion of Western dietary patterns and their consumption has been on the rise starting from the second half of the twentieth century. Additives and emulsifiers in processed foods have been linked to an accelerated progression of diet‐related non‐communicable disorders, such as inflammatory bowel disease [7], obesity [8], diabetes [9], and metabolic syndrome [10]. Ultra‐processed foods typically include one or multiple emulsifiers for texture improvement and shelf‐life extension. Accumulating evidence has shown that some emulsifiers such as carboxymethylcellulose, polysorbate‐20 and polysorbate‐80, even at relatively low doses, can induce impaired gut epithelial barrier, low‐grade inflammation, or perturbed gut microbiota composition [11, 12, 13]. These effects may contribute to the development of diet‐related chronic non‐communicable diseases.

Carrageenans (CGN, E407) are a family of linear sulfated polysaccharides derived from red seaweeds [14] and have been commonly used for decades as gelling agents, stabilisers, and thickeners in a myriad of food products, including ice cream, puddings, cocoa and chocolate products, processed meat, sauces and food supplements [15, 16]. According to a report by the European Food Safety Authority (EFSA), typical usage levels of carrageenan provided by industry range from 0.5 to 50,000 mg/kg (Table S1) [16]. It has been estimated that carrageenan intake in the United States rose approximately 170‐fold between 1972 and 2003 as a consequence of increased consumption of processed foods [17]. Exposure to carrageenan and processed Eucheuma seaweed from their use as food additives is 5.0–138.6 mg/kg bw per day for adults as reported by the EFSA Panel [16], which is in line with the doses used in our experiments.

Although carrageenan is classified and generally recognised as a safe substance by the US Food and Drug Administration (FDA), its safety or toxicity profile has been controversial [18, 19, 20]. Extensive studies on carrageenan toxicology and possible implications to intestinal health have been conducted over the past decades. It has been demonstrated that carrageenan may trigger intestinal inflammation in experiments using intestinal epithelial cells and animal models [21, 22, 23, 24, 25]. It is worth noting that three types of carrageenans have been widely used in the food industry, kappa‐ (κ‐), iota‐ (ι‐) and lambda‐ (λ‐) carrageenan. They differ in their degree of sulfation, which determines their gel‐forming ability and gel property as well as their preferential usage [15]. κ‐Carrageenan has one sulfate group in its basic disaccharide units and yields a strong firm and brittle gel used in canned meat, dessert gels, cream cheese, ice cream, processed cheese, pudding, BBQ and pizza sauces and so on; ι‐carrageenan has two sulfate groups per disaccharide and forms a soft and elastic gel used in soft gel capsules, jelly, salad dressing, cold‐prepared custards, ready to eat desserts, soy milk (Table S1). λ‐Carrageenan has three sulfates and forms viscous and non‐gelling solutions mainly used in chocolate milk, whipped cream, imitation coffee creams and so on. Differing structures and physicochemical properties of carrageenans may have varying biological effects. However, the differentiation between the different types of carrageenans (κ‐, ɩ‐ and λ‐types) in altering the gastrointestinal epithelial barrier, as well as the differential gene and protein profiles involved, has not yet been elucidated.

In the present study, the effects of food emulsifiers κ‐, ɩ‐ and λ‐carrageenans at various concentrations on the epithelial cell activation, proinflammatory and epithelial barrier integrity damaging properties were investigated using a gut‐on‐a‐chip model. The expression of differentially expressed genes and proteins was further analysed using transcriptomics and targeted proteomics to uncover the underlying mechanisms of their actions on the intestinal epithelial barrier.

## 2 Materials and Methods

### 2.1 Cell Cultures

The human colon adenocarcinoma cell line, Caco‐2, was supplied by Sigma‐Aldrich (St Louis, MO, USA). Caco‐2 cells were maintained in Eagle's Minimum Essential Medium (EMEM, ATCC, 30–2003, Manassas, VA, USA) with 10% fetal bovine serum (FBS, Sigma‐Aldrich), 1% non‐essential amino acids (Sigma‐Aldrich), 1% sodium pyruvate solution (Sigma‐Aldrich) and 2% penicillin–streptomycin (Sigma‐Aldrich) at 37°C with 5% CO_2_. Cells were passaged or seeded for experiments after reaching 80%–90% confluency in cell culture flasks.

According to the report of the EFSA panel, exposure to carrageenan and processed Eucheuma seaweed from their use as food additives is 5.0–138.6 mg/kg bw per day for adults [16], which corresponds to 350–9702 mg/70 kg bw/day. In the intestinal content of 1.5 L, ingestion of 350–9702 mg/day represents an exposure of the human intestine to 233–6468 μg/mL. Based on this, we took 5000 μg/mL as the maximum dose for the cytotoxicity test of carrageenans.

### 2.2 Apoptosis and Cytotoxicity Assays

Caco‐2 cells were seeded in 100 μL at 5000 cells/well in 96‐well plates for monolayer cultures and left overnight for attachment at 37°C in a humidified 5% CO_2_ incubator. Subsequently, κ‐, ɩ‐ and λ‐carrageenans at concentrations of 0.05, 0.5, 5, 50, 500 and 5000 μg/mL were added to treat Caco‐2 cells for 72 h. After the treatment medium was gently removed, Caco‐2 cells were washed with fresh medium and stained with 10 μg/mL Hoechst 33342 (23491‐45‐4, Sigma‐Aldrich) and 10 μg/mL propidium iodide (PI; P4864, Sigma‐Aldrich) in medium for 30 min at 37°C. The stained cells were observed under an EVOS M7000 imaging system (Thermo Fisher Scientific, Waltham, MA, USA) at 4× magnification and counted using Fiji software. The cytotoxicity was expressed as a ratio of the number of dead cells to the number of total cells. Phase‐contrast images of the monolayer cultures were taken immediately under an inverted microscope (Axio Observer A1, Zeiss, Oberkochen, Germany) at 10× magnification before the cytotoxicity determination.

Apoptosis induced by carrageenans was detected using FITC Annexin V/Dead Cell Apoptosis Kit (V13242, Invitrogen, Eugene, Oregon, USA) according to the manufacturer's instructions. Briefly, Caco‐2 cells were harvested after treatment with carrageenans at different concentrations for 72 h and centrifuged at 350 g for 7 min. The supernatant was discarded and the pellet was resuspended in 100 μL of 1 × annexin‐binding buffer. 5 μL of FITC annexin V and 1 μL of the 100 μg/mL PI working solution were added to cell suspension and incubated at room temperature for 15 min in the dark. 400 μL of 1× annexin‐binding buffer was added and the samples were analysed using an LSRFortessa flow cytometer (Becton Dickinson, San Jose, CA, USA).

### 2.3 Gut‐On‐A‐Chip Cultures

The gut‐on‐a‐chip model was established in the OrganoPlate 3‐lane 64 (6405‐400‐B, Mimetas BV, Leiden, The Netherlands), which is a microfluidic 3D tissue culture plate containing 64 independent chips. Each chip consists of one in‐gel culture channel, two perfusion channels and two phaseguides to separate the channels.

To establish the gut‐on‐a‐chip, tubules were formed by growing Caco‐2 cells against an extracellular matrix (ECM) gel. Briefly, an ECM gel (4 mg/mL) was prepared by mixing 5 mg/mL collagen‐I (Collagen Type I rat tail, ibidi GmbH, Grafelfing, Germany), 1 M HEPES (pH 7.2–7.5), 37 g/L NaHCO_3_ in an 8:1:1 ratio, kept on ice and used within 10 min. 2 μL gel was loaded into the gel inlets of each chip and the OrganoPlate was placed in a humidified incubator for 15 min to allow the gel polymerisation. After incubation, 30 μL of HBSS was added to the gel inlets to prevent the gel from drying out. The OrganoPlate was placed back in the incubator and then the other day, 20,000 Caco‐2 cells in 2 μL of complete EMEM were seeded in the right perfusion inlets, followed by adding 50 μL of complete EMEM. The OrganoPlate was placed at an angle of 75° using a holder and incubated for 3–4 h to allow Caco‐2 cells to attach to the ECM gel. After Caco‐2 cells had attached, 50 μL of complete EMEM was added to the remaining perfusion inlets and outlets. Perfusion was driven by putting the OrganoPlate on the OrganoFlow (Mimetas BV), a customised rocking platform with an inclination of 14° at an interval of 8 min for a bidirectional flow. Medium was changed every 2 days using a repeating multichannel pipette. Exposure of κ‐, ɩ‐ and λ‐carrageenans at concentrations of 5, 50, 500 and 5000 μg/mL was performed from the apical side on day 8 after cell seeding (when the tubules had formed). In the untreated group, only the medium was added to the apical compartment.

### 2.4 Transepithelial Electrical Resistance Measurement

Transepithelial electrical resistance (TER) was monitored at different time points using an automated multichannel impedance spectrometer compatible with gut‐on‐a‐chips grown in OrganoPlate 3‐lane 64 (OrganoTEER, Mimetas BV, Leiden, The Netherlands), as described previously [26]. Prior to measurement, the OrganoPlate was taken out of the incubator and put on the lab bench to equilibrate for 30 min at room temperature. The electrode board of the OrganoTEER device was placed on top of the OrganoPlate, and the electrode pairs were inserted into the medium of all perfusion inlets and outlets connecting to the apical and basal side of all chips. The OrganoTEER software generated the TER values, which are automatically normalised to Ω·cm^2^. Relative change of TER was given as a ratio to the initial TER measured prior to the carrageenan exposure.

### 2.5 Paracellular Flux Assay for Epithelial Barrier Integrity

Barrier integrity assay was performed after TER measurements. The fluorescent working solution was prepared with complete EMEM containing 0.5 mg/mL of fluorescein isothiocyanate (FITC) − dextran (150 KDa, 46946, Sigma‐Aldrich) prior to the start of the experiment. The medium was aspirated from all inlets and outlets. Subsequently, 20 μL of complete EMEM was pipetted into the inlets and outlets of the basal perfusion channel and the gel inlets. Next, 35 μL of the fluorescent working solution was added to the inlets and 15 μL to the outlets of the apical perfusion channel. After 24 h of incubation, the leakage of FITC−dextran from the apical to the basal compartment through the ECM channel was imaged using an EVOS M7000 imaging system at 4× magnification and the fluorescence intensity of the apical and basal perfusion channel was analysed using Fiji software. The paracellular flux (PF) was calculated by dividing the fluorescence intensity of the basal perfusion channel by the fluorescence intensity of the apical perfusion channel for each chip and normalising to the untreated chips.

### 2.6 Immunofluorescence Staining

Gut‐on‐a‐chips were fixed with 4% paraformaldehyde in phosphate‐buffered saline (PBS, pH 7.4) for 10 min. After washing twice with PBS and once with the wash buffer (0.1% BSA and 0.1% Triton X100 in PBS, pH 7.4), the cells were permeabilized with 0.1% Triton X100 and 0.02% SDS in PBS for 10 min. After washing with the wash buffer once for 5 min, the cells were blocked with 1% BSA and 10% normal goat serum (X0907, Dako, Glostrup, Denmark) in PBS for 45 min followed by incubation with mouse anti‐occludin antibody (1:250; CL1555; ab242202, Abcam, Cambridge, UK) and rabbit anti‐ZO‐1 antibody (1:250; 40‐2200, Invitrogen) overnight at 4°C using OrganoFlow (5 angle, 3 min interval). Alexa Fluor 488‐labelled goat anti‐mouse IgG (H + L) (1:500; A32723, Invitrogen), Alexa Fluor 546‐labelled goat anti‐rabbit IgG (H + L) (1:500; A11035, Invitrogen) and 4′, 6‐diamidino‐2‐phenylindole (DAPI) (1:5000) were added to incubate for 30 min in the dark using OrganoFlow (5 angle, 3 min interval) after washing twice for 3 min each. After incubation, the cells were washed again twice with the wash buffer for 3 min each and once with PBS for 3 min. The cells were kept in PBS at 4°C and image acquisition was performed using a Zeiss LSM 780 confocal microscope (Carl Zeiss Microscopy GmbH, Oberkochen, Germany).

### 2.7 RNA‐Sequencing

Caco‐2 cells cultured in organ‐on‐a‐chips in the OrganoPlate were fully lysed by incubating the lysis buffer for 30–60s and collected from the chips for RNA isolation using a RNeasy Plus Micro Kit (Qiagen). The quantity and quality of total RNAs were measured via 2200 Tape Station Automated Electrophoresis System (Agilent Technologies, Santa Clara, CA, USA). RNA sequencing was performed on samples with an RNA integrity number of > 9.8 using the TruSeq Stranded mRNA Sample Prep Kit (Illumina, San Diego, CA, USA) on the Illumina NovaSeq 6000. The raw sequencing reads were trimmed using BBDuk to remove adapter sequences and low‐quality bases. The trimmed reads were then mapped to the human reference genome (GRCh38.p14) using the Subread aligner. Gene expression values were quantified by feature counts implemented in the Rsubread software (Bioconductor) with annotation from Gencode release 45. Differentially expressed genes were analysed using DESeq2 (version 1.32.0). Genes with a *p* value of < 0.01 and log2 fold change of > 1 or < −1 were included in this study. Gene Ontology (GO) categories were performed using the Bioconductor package GOSeq with the Wallenius approximation.

### 2.8 NF‐κB Reporter Assay

For the NF‐κB activation assay, NF‐κB/AP‐1 reporter THP1‐XBlue (InvivoGen) monocytic cells were used. THP1‐XBlue cells were cultured in RPMI 1640 medium (Thermo Fisher Scientific) supplemented with fetal calf serum, non‐essential amino acids, sodium pyruvate, MEM vitamins, penicillin–streptomycin, normoxin and 4.5 g/L glucose. For selection, 0.2 mg/mL zeocin was used. 100,000 cells were seeded in the 96‐well plate and treated with carrageenans in a 37°C humidified incubator with 5% CO_2_ for 24 h. The QUANTI‐Blue solution was used according to the manufacturer's recommendations for the detection of NF‐κB/AP‐1 stimulation. 180 μL of QUANTI‐Blue Solution and 20 μL of cell supernatant were mixed in a 96‐well plate and incubated for 1–2 h. Optical density was measured at 655 nm with an ELISA reader.

### 2.9 Targeted Proteomics

Supernatants from the chips in the OrganoPlate were analysed using targeted proteomics via the proximity extension assay (Olink), according to the manufacturer's instructions. Immune response and inflammation panels, each of which contains 92 proteins, were used. The Olink‐generated data were preprocessed and quality‐controlled using the platform‐specific ‘Olink NPX manager’ software, which corrects background, log2 transforms and normalises all samples to an arbitrary normalised protein expression (NPX) scale.

### 2.10 Statistical Analysis

All statistical analyses and associated figures were generated using GraphPad Prism software (version 9.0) or R package. Differences among groups were analysed using the Kruskal‐Wallis test or one‐way ANOVA followed by Dunnett correction and *p* < 0.05 was considered statistically significant.

Olink data were analysed using R (version 4.2.0). Differential expression analysis of proteins was achieved using the OlinkAnalyze package (version 3.8.2). Benjamini–Hochberg's false discovery rate corrected q‐values were calculated to correct for multiple testing in all parts of the differential expression analysis. Proteins with an adjusted *p* < 0.05 were considered significantly different biomarkers. Functional analysis of the proteins identified was conducted using GO annotation, and proteins were categorised according to their biological processes.

## 3 Results

### 3.1 Carrageenans Show Dose‐Dependent Cytotoxicity on Gut Epithelial Cells

**FIGURE 1:**
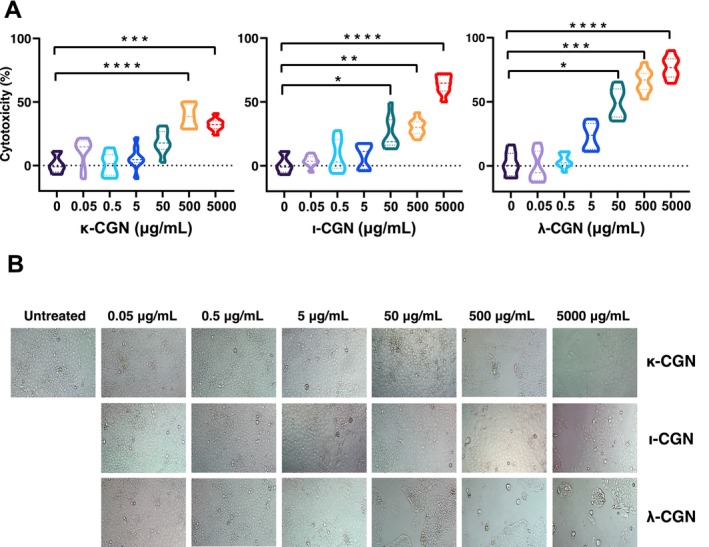
Dose‐dependent cytotoxicity of carrageenans in gut epithelial cells. (A) Cytotoxicity of κ‐, ɩ‐ and λ‐carrageenans in monolayer‐cultured Caco‐2 cells following a 72‐h exposure, assessed by an automated differential nuclear staining assay. Values were expressed as mean ± SD. (B) Representative phase‐contrast images of monolayer‐cultured Caco‐2 cells treated with different concentrations of κ‐, ɩ‐ and λ‐carrageenans for 72 h. *p < 0.05, **p < 0.01, ***p < 0.001, ****p < 0.0001, Kruskal–Wallis test with nonparametric test. ɩ‐CGN, ɩ‐carrageenan; κ‐CGN, κ‐carrageenan; λ‐CGN, λ‐carrageenan.

In vitro cytotoxicity of κ‐, ɩ‐ and λ‐carrageenans was determined using a nuclear staining assay as described previously [27]. A dose‐dependent cytotoxicity was induced by three types of carrageenans on gut epithelial cells, with significant cytotoxicity observed when exposed to κ‐carrageenan above 500 μg/mL and ɩ‐ and λ‐carrageenans above 50 μg/mL compared to the untreated group (Figure 1A). In addition, 50 μg/mL of λ‐carrageenan elicited a 49.20% ± 11.70% cytotoxicity, higher than κ‐carrageenan (18.52% ± 9.17%) and ɩ‐carrageenan (25.60% ± 12.91%) at the same concentration, indicating a more severe cytotoxic effect induced by λ‐carrageenan. Images from phase‐contrast microscopy showed cell detachment from the plate after treatment with λ‐carrageenan above 50 μg/mL and κ‐ and ɩ‐carrageenans above 500 μg/mL (Figure 1B).

We further investigated the apoptosis of monolayer‐cultured Caco‐2 cells after exposure to κ‐, ɩ‐ and λ‐carrageenans at concentrations of 0.05, 5 and 500 μg/mL using FITC annexin V/PI double staining and flow cytometric analysis. Compared to the untreated group, 5 μg/mL of λ‐carrageenan induced 1.63‐fold early (annexin V) and late (annexin V and PI) apoptosis showing cell percentages, whereas 5 μg/mL of κ‐ and ɩ‐carrageenans did not evoke obvious apoptosis (Figure S1). However, 500 μg/mL of κ‐, ɩ‐ and λ‐carrageenans induced apoptosis in 17.08% ± 0.71%, 14.53% ± 0.37% and 16.50% ± 0.23% of the cells, which were 1.67‐fold, 1.42‐fold and 1.62‐fold higher than that of the untreated group, respectively.

### 3.2 Carrageenans Induce an Impaired Epithelial Barrier

**FIGURE 2:**
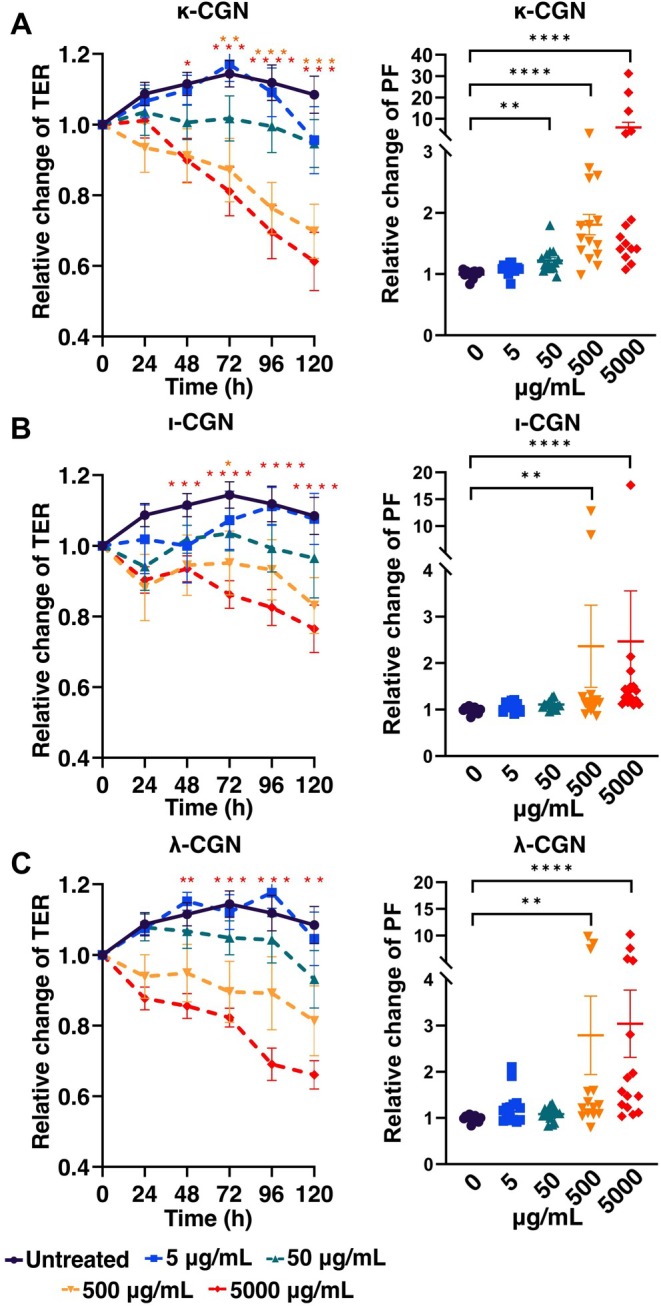
Carrageenans decreased TER and increased paracellular flux in a gut‐on‐a‐chip model. (A–C) TEER was measured every 24 h for 5 days after exposure of κ‐, ɩ‐ and λ‐carrageenans. Paracellular flux (PF) of FITC‐dextran 150 KDa from the apical side to the basal side was measured after exposure to κ‐, ɩ‐ and λ‐carrageenans for 120 h. Data are presented as mean ± SEM from three independent experiments conducted at different times. *p < 0.05, **p < 0.01, ***p < 0.001, ****p < 0.0001, Kruskal–Wallis test with nonparametric test. ɩ‐CGN, ɩ‐carrageenan; κ‐CGN, κ‐carrageenan; λ‐CGN, λ‐carrageenan.

A gut‐on‐a‐chip model was established to examine the effects of carrageenans on the gut epithelial barrier by assessing the relative changes of TER and paracellular flux. TER values were determined every 24 h after exposure of κ‐, ɩ‐ and λ‐carrageenans in a gut‐on‐a‐chip. After 120 h exposure, the paracellular flux of FITC−dextran 150 KDa from the apical side to the basal side was measured. All three carrageenans elicited a dose‐ and time‐dependent decrease of relative TER, but showed varying degrees of damage to the intestinal epithelial barrier (Figure 2). A significant decrease of relative TER was observed after treatment with 5000 μg/mL κ‐carrageenan for 48 h and 500 μg/mL κ‐carrageenan for 72 h (*p* < 0.05; Figure 2A), with a significant increase in the paracellular flux observed upon exposure to κ‐carrageenan above 50 μg/mL for 120 h (*p* < 0.01; Figure 2A). In contrast, ι‐ and λ‐carrageenans elicited a significant decrease of relative TER only when the dose reached 5000 μg/mL for 48 h, except for the treatment with 500 μg/mL ι‐carrageenan for 72 h (*p* < 0.01; Figure 2B,C). Moreover, a significant increase in the paracellular flux was observed only when exposed to ι‐ and λ‐carrageenans above 500 μg/mL (*p* < 0.01; Figure 2B,C). In summary, it was observed that κ‐carrageenan was more potent in epithelial barrier disruption compared to ι‐ and λ‐carrageenans.

### 3.3 Carrageenans Disrupt Tight Junctions

**FIGURE 3:**
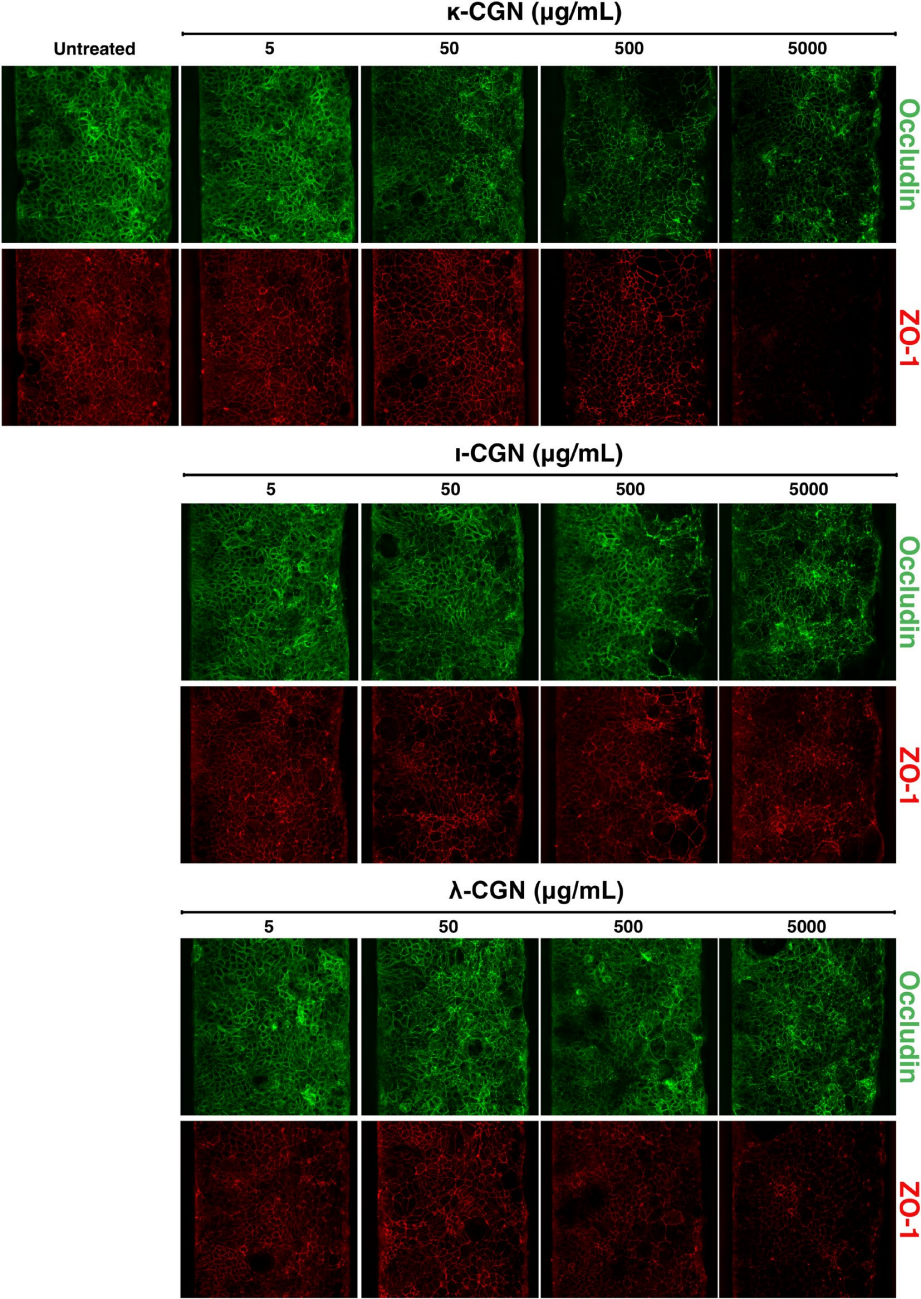
Carrageenans induced an impairment of tight junctions in a gut‐on‐a‐chip model. Immunofluorescence staining of occludin and ZO‐1 was performed after exposure of κ‐, ɩ‐ and λ‐carrageenans for 96 h and confocal immunofluorescence images were obtained. One representative of at least 3 independent experiments is shown. Staining of occludin is shown in green and ZO‐1 staining is shown in red. ɩ‐CGN, ɩ‐carrageenan; κ‐CGN, κ‐carrageenan; λ‐CGN, λ‐carrageenan.

To further understand the reduced TER and the increased paracellular flux in the gut‐on‐a‐chip model upon exposure to κ‐, ɩ‐ and λ‐carrageenans, immunofluorescence staining of two epithelial tight junction markers, occludin and ZO‐1, was performed. Both occludin and ZO‐1 showed continuous staining and well‐defined localization around cell borders in the untreated controls, indicating the presence of intact tight junctions (Figure 3). After exposure to κ‐, ɩ‐ and λ‐carrageenans at different concentrations, dose‐dependent changes in the expression levels and arrangement of occludin and ZO‐1 were observed (Figure 3). Treatments with 5 μg/mL κ‐carrageenan for 96 h did not alter the staining of occludin and ZO‐1, while discontinuous or attenuated staining of these two key proteins was observed after exposure to κ‐carrageenan starting from 50 μg/mL. However, for ɩ‐ and λ‐carrageenans, an irregular or attenuated staining of tight junction markers was observed only when their concentrations reached 500 μg/mL or more. These results demonstrated an impairment of epithelial barrier integrity, with κ‐carrageenan showing more severe effects than ɩ‐ and λ‐carrageenans, consistent with TER and paracellular flux results with the same substances.

### 3.4 Carrageenans Alter the Transcriptome of Gut Epithelial Cells

**FIGURE 4:**
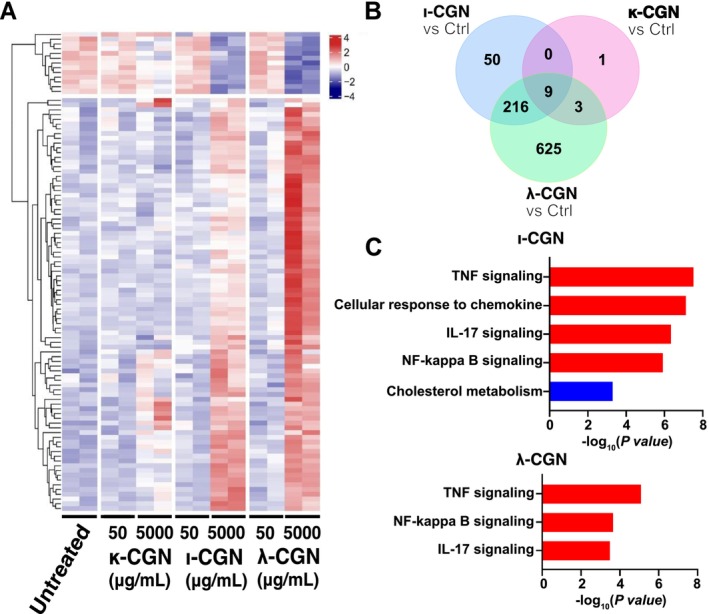
Transcriptome analysis of gut epithelial cells in response to κ‐, ɩ‐ and λ‐carrageenans in a gut‐on‐a‐chip model. (A) Heatmap of the differentially expressed genes. Each column represents a sample, and each row represents a gene. The red and blue colours indicate gene upregulation and downregulation, respectively. (B) Venn diagram illustrating the number of differentially expressed genes in response to κ‐, ɩ‐ and λ‐carrageenans at the concentration of 5000 μg/mL after 24 h. (C) Significantly upregulated and downregulated genes in response to ɩ‐ and λ‐carrageenans at 5000 μg/mL were analysed for pathway enrichment according to Kyoto Encyclopedia of Genes and Genomes (KEGG) and Gene Ontology (GO) biological process. The red and blue bars indicate up and down regulation, respectively. ɩ‐CGN, ɩ‐carrageenan; κ‐CGN, κ‐carrageenan; λ‐CGN, λ‐carrageenan.

RNA sequencing was performed to analyse the differential gene expression of gut epithelial cells after a 24 h exposure to κ‐, ɩ‐ and λ‐carrageenans in the gut‐on‐a‐chip model. When exposed to three types of carrageenans, the up‐ and down‐regulation of differentially expressed genes in gut epithelial cells showed a dose‐dependent effect (Figure 4A).

Additionally, a distinct difference in gene expression was observed in cells exposed to κ‐, ɩ‐ and λ‐carrageenans (Figure 4B). There was a total of 275 differentially expressed genes in response to 5000 μg/mL ɩ‐carrageenan and 853 specifically expressed genes in response to 5000 μg/mL λ‐carrageenan compared with the untreated group. However, 5000 μg/mL κ‐carrageenan induced differential expression of only 13 genes compared to the untreated group, of which 12 differentially expressed genes were also present in response to the other two carrageenans (Figure 4B). Although the change in epithelial barrier was more visible by κ‐carrageenan, the results suggested that there was a lack of a unique mechanism of action in terms of mRNA expression changes for κ‐carrageenan.

**FIGURE 5:**
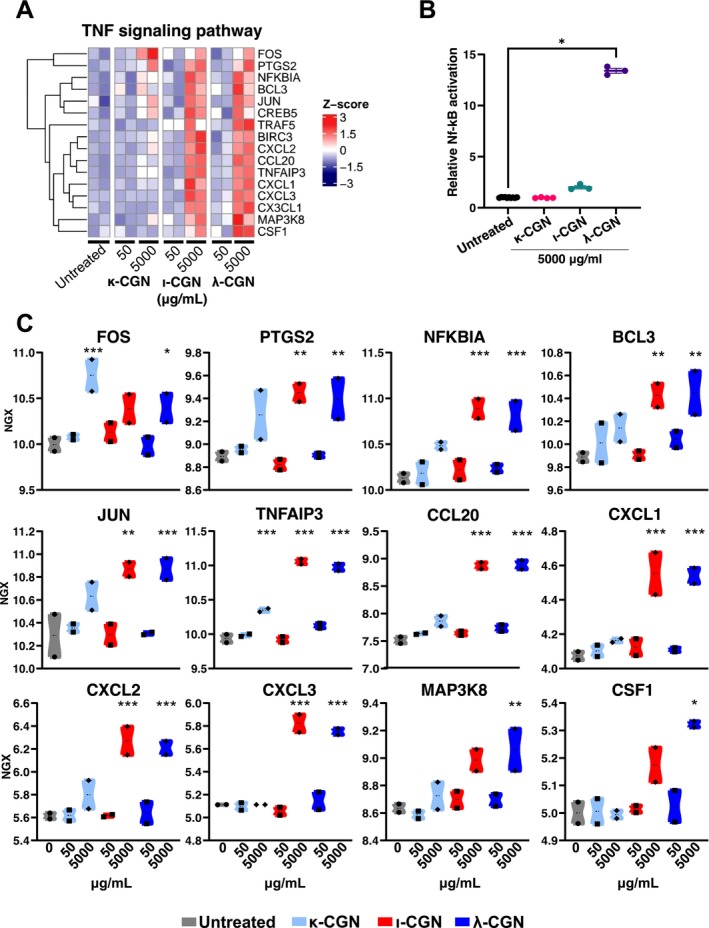
Differentially expressed genes in response to κ‐, ɩ‐ and λ‐carrageenans in monolayer cultured Caco‐2 cells. (A) Heatmap of the differentially expressed genes. Genes involved in TNF signalling pathway that were significantly altered in response to κ‐, ɩ‐, λ‐carrageenan at the concentration of 5000 μg/mL vs. unexposed. Each column represents a sample, and each row represents a gene. The red and blue colours indicate gene upregulation and downregulation, respectively. (B) Relative NfkB activation in response to κ‐, ɩ‐ and λ‐carrageenans at 5000 μg/mL were analysed. (C) Violin plots showing differentially expressed genes in response to κ‐, ɩ‐ and λ‐carrageenans at the concentrations of 50 and 5000 μg/mL. *p < 0.05, **p < 0.01, ***p < 0.001. NGX, normalised gene expression. ɩ‐CGN, ɩ‐carrageenan; κ‐CGN, κ‐carrageenan; λ‐CGN, λ‐carrageenan.

Differences in gene expression were analysed for KEGG and GO term enrichment to identify biological pathways affected by carrageenans. 5000 μg/mL ɩ‐carrageenan significantly upregulated the tumour necrosis factor (TNF) signalling, interleukin‐17 (IL‐17) signalling, cellular response to chemokine and NF‐kappa B (NF‐κB) signalling pathways, while downregulated cholesterol metabolism (Figure 4C). At the same concentration, differentially expressed genes induced by λ‐carrageenan were primarily associated with the TNF signalling pathway, IL‐17 signalling pathway and NF‐κB signalling pathway (Figure 4C). These pathways were closely linked to the activation of innate immune responses. Heatmap analyses of genes associated with these pathways were presented in Figure 5A and Figure S2. Notably, λ‐carrageenan at 5000 μg/mL markedly enhanced NF‐κB activation compared to the untreated group (Figure 5B). Figure 5C and Figure S3 demonstrated specific changes in signalling pathway‐related genes. TNF alpha induced protein 3 (TNFAIP3), which acts as a negative feedback regulator of inflammation and immunity, showed a significant increase in expression under the action of three types of carrageenans at a concentration of 5000 μg/mL. The chemokines including C‐X‐C motif chemokine ligand 1 (CXCL1), CXCL2, CXCL3, CXCL8, C‐C motif chemokine ligand 20 (CCL20) and C‐X3‐C motif chemokine ligand 1 (CX3CL1), anti‐inflammatory factors including nuclear factor‐kappa‐B‐inhibitor alpha (NFKBIA), zinc finger CCCH‐Type containing 12A (ZC3H12A) and CAMP responsive element binding protein 5 (CREB5) and proinflammatory factors prostaglandin‐endoperoxide synthase 2 (PTGS2), inhibitor of nuclear factor kappa B kinase subunit epsilon (IKBKE), RELB proto‐oncogene (RELB) and BCL3 transcription coactivator (BCL3) were significantly upregulated under the effect of 5000 μg/mL ɩ‐carrageenan or λ‐carrageenan, whereas κ‐carrageenan at the same concentration did not induce the same changes. Moreover, the proinflammatory factors such as TNF receptor‐associated factor 5 (TRAF5) and mitogen‐activated protein kinase 8 (MAP3K8) were only significantly upregulated at 5000 μg/mL λ‐carrageenan. In addition, the effects of 5000 μg/mL ɩ‐ and λ‐carrageenans resulted in a significant downregulation of apolipoprotein C3 (APOC3), ATP binding cassette subfamily A member 1 (ABCA1), apolipoprotein B (APOB), cytochrome P450 family 27 subfamily A member 1 (CYP27A1) and sterol O‐acyltransferase 2 (SOAT2) (Figure 5C), reflecting impaired lipid absorption and transport functions in gut epithelial cells.

To more intuitively demonstrate the effects of three carrageenans on gene expression in gut epithelial cells, we performed a pairwise comparative analysis and visualised the results using volcano plots (Figure S4). λ‐carrageenan treatment produced more differentially expressed genes compared to κ‐ and ɩ‐carrageenan treatment. Overall, λ‐carrageenan had the strongest pro‐inflammatory transcriptional regulation specificity, followed by ɩ‐carrageenan, with dual effects of inflammation and metabolic inhibition; in contrast, κ‐carrageenan had the least effect on the transcriptome. However, κ‐carrageenan at a concentration of 5000 μg/mL could still cause significant damage to the epithelial barrier, suggesting that its pathological effects might be mainly due to direct structural damage.

### 3.5 Targeted Proteomics of Gut Epithelial Cells in Response to Different Carrageenans

**FIGURE 6:**
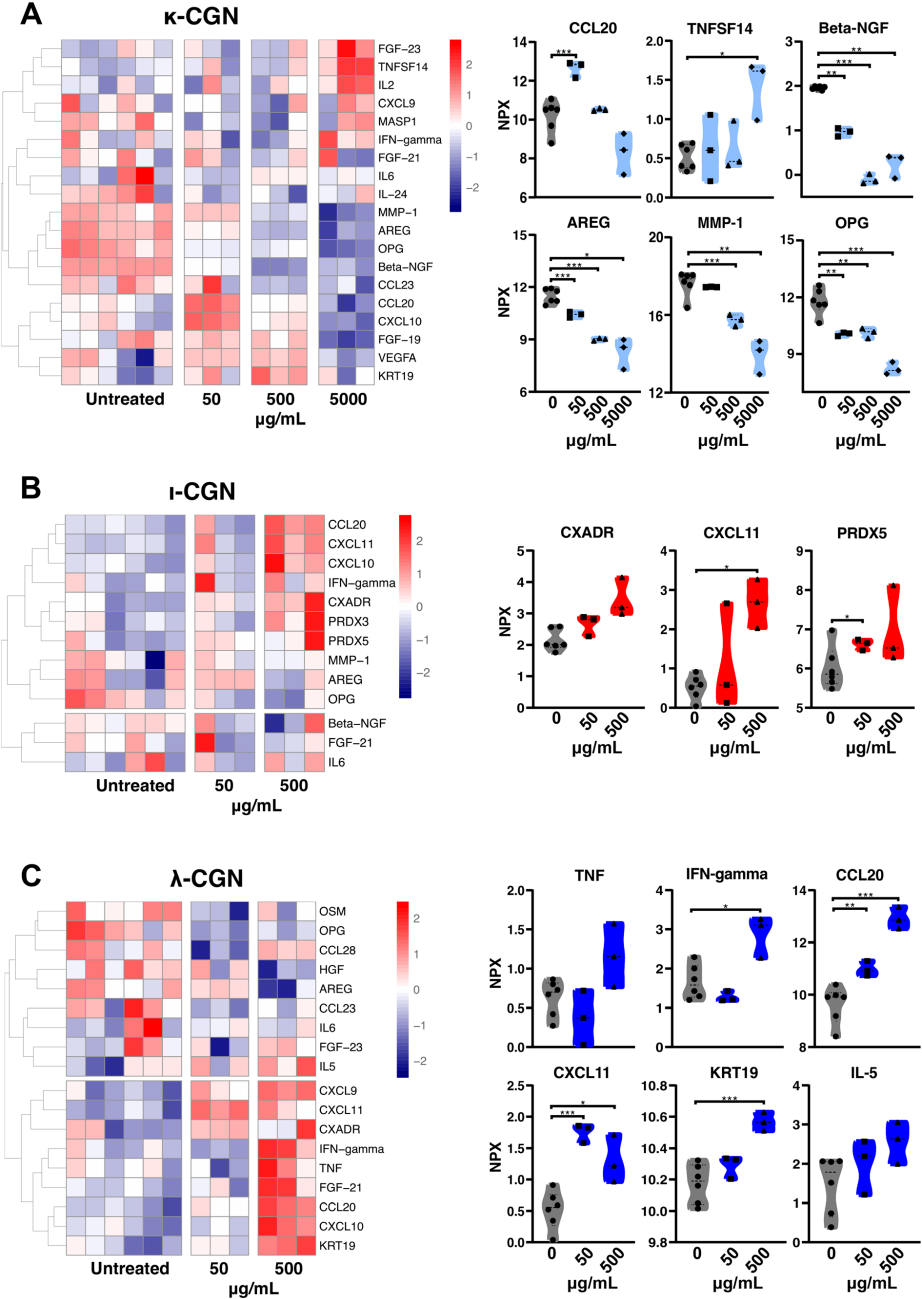
Differentially regulated proteins in response to κ‐, ɩ‐ and λ‐carrageenans in a gut‐on‐a‐chip model. (A) Heatmap and violin plots of differentially expressed proteins in response to κ‐carrageenan at the concentrations of 50, 500 and 5000 μg/mL. (B) Heatmap and violin plots of differentially expressed proteins in response to ɩ‐carrageenan at the concentrations of 50 and 500 μg/mL. (C) Heatmap and violin plots of differentially expressed proteins in response to λ‐carrageenan at the concentrations of 50 and 500 μg/mL. Each column represents a sample, and each row represents a protein. The red and blue colours indicate protein upregulation and downregulation, respectively. *p < 0.05, **p < 0.01, ***p < 0.001. NPX, normalised protein expression. ɩ‐CGN, ɩ‐carrageenan; κ‐CGN, κ‐carrageenan; λ‐CGN, λ‐carrageenan.

The proximity extension assay was performed with the inflammation and immune response panels to analyse the difference in protein expression after 48 h of exposure to κ‐, ɩ‐ and λ‐carrageenans at concentrations of 50, 500 and 5000 μg/mL. Cell lysates treated with 5000 μg/mL of ɩ‐ and λ‐carrageenans were not able to be analysed by the assay due to their reactivity with the matrix. Changes in protein expression related to immune response and inflammation were focused, as shown in Figure 6.

In agreement with the transcriptome results, changes in protein expression with the κ‐carrageenan at 500 μg/mL doses were much less compared to ɩ‐ and λ‐carrageenans. The chemokines CCL20 and CXCL10 were significantly upregulated by 50 μg/mL κ‐carrageenan, while upregulation of cytokeratin 19 (KRT19) was observed when the dose of κ‐carrageenan reached 500 μg/mL (Figure 6A). The expression of tumour necrosis factor superfamily member 14 (TNFSF14) showed an increasing trend as the concentration of κ‐carrageenan increased, while κ‐carrageenan downregulated the expression of beta nerve growth factor (beta‐NGF), amphiregulin (AREG), matrix metalloproteinase‐1 (MMP‐1) and osteoprotegerin (OPG) in a dose‐dependent manner. In contrast, only CCL20, CXCL11 and peroxiredoxin 5 (PRDX5) showed altered expression levels by the action of ɩ‐carrageenan (Figure 6B). λ‐Carrageenan induced more significant changes in protein expression than ɩ‐carrageenan (Figure 6C). The expression of CCL20 and CXCL11 significantly increased when the cells were exposed to λ‐carrageenan at concentrations of 50 and 500 μg/mL, while interferon‐gamma (IFN‐γ) and KRT19 were significantly upregulated by 500 μg/mL λ‐carrageenan. TNF and IL5 also showed an increasing trend when cells were exposed to λ‐carrageenan. λ‐Carrageenan showed the most extensive pro‐inflammatory and immune activation network at the protein expression level, whereas ɩ‐darrageenan was limited to chemokines and oxidative stress regulation. Although minimally perturbed, κ‐carrageenan selectively inhibited repair proteins (beta‐NGF, MMP‐1, etc.), indirectly confirming that its disruption of epithelial homeostasis might be independent of transcriptional regulation.

## 4 Discussion

Ultra‐processed foods and food additives are key features of the change in food supply, driven by changes in population demographics, urbanisation and employment patterns [28]. The most commonly used food additives are those with emulsifying and thickening properties, also known as food emulsifiers [29]. Accumulating evidence links food emulsifier usage/exposure to an increase in chronic non‐communicable inflammatory diseases, particularly in relation to food emulsifiers after the mid‐twentieth century [11, 30]. An impaired gastrointestinal epithelial barrier function is one of the key risk factors in the pathogenesis of these diseases. The present study provided direct evidence for the detrimental effects of food emulsifiers κ‐, ɩ‐ and λ‐carrageenans on gut epithelial integrity and inflammation. Using Caco‐2 cell cultures and a gut‐on‐a‐chip model, we demonstrated that κ‐, ɩ‐ and λ‐carrageenans caused cytotoxicity, gut epithelial barrier damage and epithelial inflammation in a dose‐dependent manner and directly impaired barrier integrity of gut epithelial cells, with similar and somewhat different underlying mechanisms. Currently, carrageenan is used more often in the production of dairy products, juice drinks, ice cream, sugar products, meat products and beer. The reported use levels of carrageenans provided by industry are shown in Table S1, and it is not difficult to see that there are still no clear regulations regarding the maximum permissible level (MPL) of carrageenan in many food products [16]. Hence, we chose to refer to the report of the EFSA panel on the dose of carrageenan (5.0–138.6 mg/kg bw per day) to adults exposed to carrageenan and processed Eucheuma seaweed as the basis for setting carrageenan concentrations in this experiment. As an important food emulsifier, carrageenan has a long history of use in traditional food processing across Asia and Europe [31]. Commercial production of its powdered form began in the early 19th century and was primarily used as a stabiliser in ice cream and chocolate milk to prevent phase separation and improve texture [32]. Carrageenan was granted generally regarded as safe by the United States in 1959, defining it as a substance generally recognised as safe [33]. Although carrageenan has long been safely consumed in traditional diets, modern industrial applications, including changes in processing techniques, increases in exposure levels and its incorporation into complex food matrices, may endow it with biological activity that differs from traditional contexts [31, 34]. After carrageenan was found to have the ability to inhibit the activity of digestive enzymes in 1967 [35], numerous studies have since emerged showing its detrimental effects on the intestinal tract. It has been previously reported that κ‐carrageenan may exacerbate pathogen‐induced intestinal inflammation [36]. A similar phenomenon has been reported with λ‐carrageenan [37]. Some studies on animal models have indicated that the intake of carrageenan (average daily intake between 1.7 and 41.7 mg/kg) encourages colonic inflammation, exhibiting the development of inflammatory infiltrates and clinical evidence of colitis [19]. Gaps existed in terms of the type and dose of carrageenan's direct effect on the degree of damage and inflammation of the epithelial barrier.

The ‘Epithelial Barrier Theory’ emphasises that factors damaging the epithelial barrier lead to a vicious cycle characterised by epithelitis, migration of microbiota to the subepithelial space and dysbiosis [1]. The severity of the pathology induced by these detrimental factors is dependent upon the substances, the duration of exposure, the dose and the type of affected tissues. Our data also provided support for the argument, as mentioned earlier. The results showed that κ‐, ɩ‐ and λ‐carrageenans induced dose‐dependent cytotoxicity, with λ‐carrageenan showing a more severe cytotoxic effect on gut epithelial cells. All three carrageenans negatively affected the intestinal epithelial barrier function, inducing epithelial barrier damage, while ɩ‐ and λ‐carrageenans induced a more severe pro‐inflammatory response in transcriptome and proteome analyses. The occurrence of the above phenomenon may be attributable to the different number of sulphate groups in carrageenans. κ‐, ι‐ and λ‐Carrageenans have one, two and three sulfate groups in their basic disaccharide units, respectively [38]. Sulfate is reported to inhibit the activity of the sulfatases, which induce an elevation in the level of total sulfated glycosaminoglycans (GAGs) [39, 40, 41]. GAGs, linear carbohydrates such as heparan sulfate and hyaluronan, play a crucial role in molecular and cellular events of inflammation, including cell‐matrix interactions and activation of chemokines [42, 43]. Carrageenan has been reported to reduce sulfatase activity and increase sulfated GAGs in human epithelial cells, with λ‐carrageenan having the greatest inhibitory effect on the activity of sulfatase enzymes [44]. Accordingly, differences in the number of their own sulphate groups may play a role in different proinflammatory and cytotoxic effects induced by κ‐, ɩ‐ and λ‐carrageenans.

In the pathway analysis of the transcriptome data, both 5000 μg/mL ɩ‐carrageenan and λ‐carrageenan affected the TNF signalling pathway, IL‐17 signalling pathway and NF‐κB signalling pathway. In addition, the pathway involving cellular response to chemokine was upregulated as an additional proinflammatory pathway. NF‐κB is a master regulator of the innate immune response and is involved in the release of many pro‐inflammatory cytokines. At a concentration of 5000 μg/mL, ɩ‐carrageenan and λ‐carrageenan were more effective in increasing the expression levels of cytokines and chemokines. The significant upregulation of chemokines and proinflammatory factors indicated the induction of a strong inflammatory response in gut epithelial cells. Meanwhile, the upregulation of anti‐inflammatory factors, such as TNFAIP3, NFKBIA, ZC3H12A and CREB5, suggested that a negative feedback response in cells has been activated to downregulate the overactivation of inflammatory signals. Differential gene expression in the NF‐kB signalling pathway indicated that carrageenans not only activated classical pathways, such as the upregulation of NFKBIA, to regulate inflammatory responses but also potentially modulated cell survival and immune responses through non‐classical pathways, including the upregulation of RELB [45]. Nuclear factor kappa B subunit 2 (NFKB2) has been demonstrated to enhance the expression of RelA‐driven proinflammatory genes in the intestinal epithelium, thereby exacerbating inflammatory cell infiltration and colon tissue damage [46]. Notably, λ‐carrageenan significantly upregulated the pro‐inflammatory factors TRAF5 and MAP3K8, important signalling proteins in the non‐canonical NF‐κB pathway indicating a broader and stronger induction of the inflammatory response, which might play a role in further intensified inflammatory damage to gut epithelial cells. Previous studies demonstrated that exposure of human intestinal epithelial cells to carrageenan might result in the upregulation of Bcl10 and both cytoplasmic and nuclear NF‐κB, along with the activation of the IL‐8 promoter. Notably, Bcl10 possessed a caspase‐recruitment domain to the susceptibility gene NOD2/CARD15 associated with Crohn's disease. Therefore, carrageenan might contribute significantly to the environmental‐genetic interplay in inflammatory bowel disease by activating pathways linked to genetic susceptibility [47]. Furthermore, carrageenan could also trigger inflammation via the TLR4 pathway, leading to the production of pro‐inflammatory cytokines and the recruitment of immune cells to exposed sites [48, 49]. Activation of TLR4 typically leads to the activation of downstream signalling pathways, of which the NF‐κB pathway is one of the most important. Overall, this inflammatory microenvironment might have led to impaired intestinal epithelial barrier function and contributed to the tissue infiltration of immune cells (such as neutrophils, eosinophils and lymphocytes), further exacerbating the inflammatory response. Cholesterol metabolism was downregulated by 5000 μg/mL ɩ‐carrageenan. The significant downregulation of cholesterol metabolism‐related genes might play a role in the disruption of lipid homeostasis, thereby affecting cell membrane integrity and barrier function [50]. Particularly, the changes in ABCA1 and APOB suggested that carrageenan inhibited cholesterol synthesis and transport, compromising cell membrane integrity and function. This disruption interfered with cell signalling and epithelial barrier function [51].

The activation of the TNF‐signalling pathway was primarily mediated by the TNF‐α/TNFR1 axis, which subsequently triggered the NF‐κB‐signalling pathway. This cascade promoted the expression of proinflammatory cytokines (e.g., IL‐1 and IL‐6) and chemokines (e.g., CXCL1, CXCL2 and CXCL3) [52, 53]. Additionally, the upregulation of chemokines such as CCL20, CXCL2 and CXCL8 may facilitate the recruitment of neutrophils and monocytes, further exacerbating local inflammatory responses [54]. ɩ‐ and λ‐Carrageenans can additionally amplify the inflammatory response by upregulating the IL‐17 signalling pathway [55]. Notably, the upregulation of PTGS2 promoted prostaglandin synthesis and enhanced the release of inflammatory mediators [56]. In summary, carrageenan synergistically activated the TNF, IL‐17 and NF‐κB signalling pathways, significantly upregulating key genes such as TNFAIP3, baculoviral IAP repeat containing 3 (BIRC3), CXCL3, NFKBIA, CXCL2 and CCL20. This network of synergistic events may lead to the release of proinflammatory cytokines and chemokines, driving inflammatory responses and recruiting immune cells to exacerbate local inflammation.

The analysis of differential proteins revealed that the presence of κ‐carrageenan not only activated the immune system but also caused damage to intestinal epithelial tissue, as a significant upregulation of two chemokines, CCL20 and CXCL10, was observed. In general, CCL20 promotes the recruitment of Tregs and Th17 cells [57, 58]. CXCL10 is considered to be the preferred chemoattractant for the activation of T‐cells, especially Th1 cells [59]. Studies on inflammatory bowel disease have demonstrated that both CCL20 and CXCL10 have been associated with inflammatory bowel disease [60]. On the other hand, the expression of beta‐NGF, AREG, MMP‐1 and OPG is important for wound healing and tissue remodelling together with suppression of inflammation [61, 62, 63, 64]. All of the above proteins were significantly altered by κ‐carrageenan. λ‐Carrageenan induced alterations in four chemokines and tended towards a Th1‐type inflammatory response due to the upregulation we found in the expression levels of IFN‐γ, a prototypic Th1 cytokine. In addition, IFN‐γ was one of the most highly upregulated cytokines in Crohn's disease (CD) and ulcerative colitis (UC), as well as in related mouse models [65].

Carrageenan has been patented around the 1930s and is widely used as a food additive. It is defined as a substance that is generally regarded as safe. However, the safety of carrageenan has been a hot topic of discussion in the scientific community from the past to the present. Our current understanding of the ‘Epithelial Barrier Theory’ suggests that it is nowadays essential to redefine the ‘toxicity’ of carrageenan from a new perspective. The licences of these substances have been given by lethal dose defining experiments in mice before the 1980s. However, recent studies, including this one, bring a new concept of chronic molecular toxicity in response to the exposure of these substances, which may play a role in the development of gastrointestinal inflammatory diseases. Overall, this study provided new insights into the potential mechanisms of intestinal epithelial barrier damage by κ‐, ɩ‐ and λ‐carrageenans. The results suggested that κ‐, ɩ‐ and λ‐carrageenans negatively influenced the maintenance of intestinal epithelial barrier function, inducing epithelial barrier damage, while ɩ‐ and λ‐carrageenans induced a more severe pro‐inflammatory response. As a mature in vitro intestinal model with high flexibility, high repeatability and low cost, the differentiated Caco‐2 cell model has been applied to a variety of intestinal studies including intestinal absorption, intestinal transport, intestinal metabolism, intestinal barrier, intestinal immunity and intestinal adhesion [66]. However, the Caco‐2 cell monolayer model does possess limitations in fully simulating the complex in vivo environment of the human intestine, particularly lacking the crucial mucus layer structure [67]. This mucus layer where dietary components (such as carrageenan) first come into contact and may interact before reaching the epithelium. This may lead to discrepancies between the results of this experiment and actual human conditions. To support these results based on Caco‐2 cells and intestinal organ‐on‐a‐chip models, in vivo data are urgently needed to assess the health hazards of different carrageenan types and other emulsifiers and additives used in ultraprocessed food. These findings not only involve humans but there is a very common usage of emulsifiers in the processed food of domestic animals, including carrageenans [68]. By understanding the inflammatory pathways induced by carrageenan, we can explore treating or mitigating its inflammatory effects and provide guidance for safe processing in the food industry.

## Author Contributions

N.S., D.Y., Y.P., I.O., R.D., M.A., K.C.N. and C.A.A. contributed to conceptualization. Methodology was performed by N.S., D.Y., Y.P., B.R., X.B., M.L., I.O. and C.A.A. Data collection was conducted by N.S., D.Y., Y.P. and S.J. Formal analysis was carried out by H.B. and S.A. The original draft was written by N.S. Review and editing of the manuscript were performed by N.S., D.Y., Y.P., B.R., S.J., X.B., M.L., I.O. and C.A.A. Supervision was provided by D.Y., Y.P., B.R., M.A., R.D., K.C.N. and C.A.A. All authors have read and agreed to the published version of the manuscript.

## Funding

This work was supported by the Swiss National Science Foundation (SNF) and the EU Project, ‘Disrupting Noxious Synergies of Indoor Air Pollutants and their Impact in Childhood Health and Wellbeing, using Advanced Intelligent Multisensing and Green Interventions’ (SynAir‐G).

## Ethics Statement

The authors have nothing to report.

## Conflicts of Interest

M.A. has received research grants from the Swiss National Science Foundation, Bern; research grant from Stanford University; Leading House for the Latin American Region, Seed Money Grant. She is the Scientific Advisory Board member of Stanford University Sean Parker Asthma Allergy Center, CA; Advisory Board member of LEO Foundation Skin Immunology Research Center, Copenhagen; and Scientific Co‐Chair of World Allergy Congress (WAC) Istanbul, 2022, Scientific Programme Committee Chair, EAACI. K.C.N. currently reports grants from National Institute of Allergy and Infectious Diseases (NIAID), National Heart, Lung and Blood Institute (NHLBI), National Institute of Environmental Health Sciences (NIEHS); Stock options from IgGenix, Seed Health, ClostraBio, Cour, Alladapt; Consultant for Excellergy, Red tree ventures, Regeneron and IgGenix; Co‐founder of Alladapt, Latitude and IgGenix; National Scientific Committee member at Immune Tolerance Network (ITN) and National Institutes of Health (NIH) clinical research centres; patents include, ‘Mixed allergen composition and methods for using the same’, ‘Granulocyte‐based methods for detecting and monitoring immune system disorders’ and ‘Methods and Assays for Detecting and Quantifying Pure Subpopulations of White Blood Cells in Immune System Disorders’. C.A.A. has received research grants from the Swiss National Science Foundation, European Union (EU CURE, EU Syn‐Air‐G), Novartis Research Institutes (Basel, Switzerland), Stanford University (Redwood City, Calif), Seed Health (Boston, USA) and SciBase (Stockholm, Sweden); is the Co‐Chair for EAACI Guidelines on Environmental Science in Allergic diseases and Asthma; is on the Advisory Boards of Sanofi/Regeneron (Bern, Switzerland, New York, USA), Stanford University Sean Parker Asthma Allergy Center (CA, USA), Novartis (Basel, Switzerland), Glaxo Smith Kline (Zurich, Switzerland), Bristol‐Myers Squibb (New York, USA), Seed Health (Boston, USA) and SciBase (Stockholm, Sweden); and is the Editor‐in‐Chief of Allergy. I.O. is chair of the EAACI Epithelial Cell Biology Working Group. N.S. has received grants from CSC scholarship program of China (No. 202008210164). R.D. is a non‐executive Board Director at Seed Health Inc. Y.P., H.B., S.A., X.B., M.L., S.J., B.R. and D.Y. declare no relevant conflicts of interest.

## Supporting information


**Data S1:** cea70266‐sup‐0001‐Supinfo.pdf.

## Data Availability

The data that support the findings of this study are available from the corresponding author upon reasonable request.
