# Supplementary material for: Effects of Common Food Additives Kappa‐, Iota‐ and Lambda‐Carrageenans on Intestinal Epithelial Cell Activation and Barrier Disruption
Source: Clin Exp Allergy. 2026 Apr 6;56(7):769–83. doi: 10.1111/cea.70266 (PMC13327219; doi:10.1111/cea.70266)
Supplement: Supplementary file 1 — Data S1: cea70266‐sup‐0001‐Supinfo.pdf. [file CEA-56-769-s001.pdf]

Supplementary Table 1 Summary of reported usag levels (mg/kg or mg/L as appropriate) of carrageenan provided by industry[1]

Food category name	E number/Group	MPL	Provided by	Mean of typical usage levels	Minimum of typical usage levels	Maximum of typical usage levels	Maximum usage levels
Flavoured fermented milk products including heat treated products	Group I	QS	FDE (2014)	10800	10800	10800	22256
			Intertek Scientific & Regulatory Consultancy (2014)	1075	150	2000	3000
			Rudolf Wild GmbH & Co. KG (2014)	870	780	960	6300
Dehydrated milk as defined by Directive 2001/114/EC	E 407	QS	Intertek Scientific & Regulatory Consultancy (2014)	253	50	912	1368
Unflavoured pasteurised cream (excluding reduced fat creams)	E 407	QS	Intertek Scientific & Regulatory Consultancy (2014)	773	140	2000	2500
			Intertek Scientific & Regulatory Consultancy (2014)	50	50	50	100
Unflavoured live fermented cream products and substitute products with a fat content of less than 20%	E 407	QS	Intertek Scientific & Regulatory Consultancy (2014)	95	50	140	175
Other creams	Group I	QS	FDE (2014)	1584	200	3602	7000
			Intertek Scientific & Regulatory Consultancy (2014)	645	50	2000	2500
Unripened cheese excluding products falling in category 16	Group I	QS	Intertek Scientific & Regulatory Consultancy (2014)	1750	1750	1750	3500
Processed cheese	Group I	QS	FDE (2014)	2500	2500	2500	5000
			Intertek Scientific & Regulatory Consultancy (2014)	3355	1400	7500	10000
Cheese products (excluding products falling in category 16)	Group I	QS	Intertek Scientific & Regulatory Consultancy (2014)	350	350	350	1400
Dairy analogues, including beverage whiteners	Group I	QS	FDE (2013)	1081	22	6959	6959
			FDE (2014)	450	350	525	1000
			Intertek Scientific & Regulatory Consultancy (2014)	1440	140	4000	14000
			Intertek Scientific & Regulatory Consultancy (2014)	2667	1000	5000	10000
Other fat and oil emulsions including spreads as defined by Council Regulation (EC) No 1234/2007 and liquid emulsions	Group I	QS	Intertek Scientific & Regulatory Consultancy (2014)	2800	2800	2800	4200
Edible ices	Group I	QS	BABBI Confectionery Industry (2014)	600	600	600	740
			Interested Party I (2014)	1650	300	3000	10000
			FDE (2013)	232	17	1736	2090
			FDE (2014)	2748	205	4500	4500
			FDE (2014)	2052	180	6000	6000
			Intertek Scientific & Regulatory Consultancy (2014)	148	100	180	700
			Intertek Scientific & Regulatory Consultancy (2014)	150	100	200	500
			Rudolf Wild GmbH & Co. KG (2014)	750	750	750	2000
Dried fruits and vegetables	Group I	QS	Intertek Scientific & Regulatory Consultancy (2014)	5000	5000	5000	10000
Fruit and vegetable preparations excluding compote	Group I	QS	FDE (2013)	0.6	0.6	0.6	0.6
			FDE (2014)	450	450	450	1000

			Intertek Scientific & Regulatory Consultancy (2014)	4333	3000	5000	10000
			Intertek Scientific & Regulatory Consultancy (2014)	3400	200	5000	10000
Jam, jellies and marmalades and sweetened chestnut puree as defined by Directive 2001/113/EC	E 407	10000 mg/kg	EUROGUM A/S (2014)	5000	5000	5000	10000
			Intertek Scientific & Regulatory Consultancy (2014)	3750	3500	4000	8000
Processed potato products	Group I	QS	FDE (2013)	21	21	21	21
Cocoa and Chocolate products as covered by Directive 2000/36/EC	Group I	QS	FDE (2014)	288	288	288	1913
			Intertek Scientific & Regulatory Consultancy (2014)	963	150	3000	5000
			Intertek Scientific & Regulatory Consultancy (2014)	200	200	200	300
Other confectionery including breath refreshening microsweets	Group I	QS	FDE (2014)	414	414	414	980
			Intertek Scientific & Regulatory Consultancy (2014)	12000			15000
			Intertek Scientific & Regulatory Consultancy (2014)	8500			25000
			Rudolf Wild GmbH & Co. KG (2014)	3500	3500	3500	12000
Chewing gum	Group I	QS	International Chewing Gum Association (2013)	4000	4000	4000	15000
Decorations, coatings and fillings, except fruit-based fillings covered by category 4.2.4	Group I	QS	FDE (2014)	1918			10000
			Intertek Scientific & Regulatory Consultancy (2014)	3380			7000
			Intertek Scientific & Regulatory Consultancy (2014)	1800			10000
Pre-cooked or processed cereals	Group I	QS	FDE (2014)	295	295	295	1040
Bread and rolls	Group I	QS	FDE (2014)	396	396	396	529
			Intertek Scientific & Regulatory Consultancy (2014)	1000	1000	1000	2000
Fine bakery wares	Group I	QS	Interested Party I (2014)	20	20	20	40
			FDE (2013)	2102	2102	2102	3528
			FDE (2014)	6789	366	10000	20000
			FDE (2014)	1896	500	3291	3291
			Rudolf Wild GmbH & Co. KG (2014)	3000	3000	3000	3900
Meat preparations as defined by Regulation (EC) No 853/2004	E 407/ E 407a	QS	EUROGUM A/S (2014)	5000	5000	5000	20000
			FDE (2014)	30	30	30	11250
Non-heat-treated processed meat	Group I	QS	Associazione Industriali delle Carni e dei Salumi (2014)	1000	1000	1000	1000
			Fabricante Embutidos del centro SA (España) (2014)	3			4
			FDE (2014)	125	125	125	1750
			Intertek Scientific & Regulatory Consultancy (2014)	480			600
			Intertek Scientific & Regulatory Consultancy (2014)	3000	3000	3000	5000
Heat-treated processed meat	Group I	QS	Associazione Industriali delle Carni e dei Salumi (2014)	1460	0	2200	2200
			EUROGUM A/S (2014)	5000	5000	5000	20000
			Fabricante Embutidos del centro SA (España) (2014)	7	6	10	10
			FDE (2013)	5	5	5	5
			FDE (2014)	714	127	1375	1973
			Intertek Scientific & Regulatory Consultancy (2014)	3108	2000	5000	8000
			Intertek Scientific & Regulatory Consultancy (2014)	2200	1000	3000	5000

Casings and coatings and decorations for meat	Group I	QS	Intertek Scientific & Regulatory Consultancy (2014)	3500	1000	6000	8000
Processed fish and fisheries products including molluscs and crustaceans	Group I	QS	Intertek Scientific & Regulatory Consultancy (2014)	2050	300	5000	8000
Fish roe	Group I	QS	Intertek Scientific & Regulatory Consultancy (2014)	2800	2800	2800	5600
Processed eggs and egg products	Group I	QS	Intertek Scientific & Regulatory Consultancy (2014)	100	100	100	500
Processed eggs and egg products	Group I	QS	Intertek Scientific & Regulatory Consultancy (2014)	5000	5000	5000	5000
Other sugars and syrups	Group I	QS	Intertek Scientific & Regulatory Consultancy (2014)	350	350	350	700
Seasonings and condiments	Group I	QS	Intertek Scientific & Regulatory Consultancy (2014)	600	600	600	1000
Soups and broths	Group I	QS	FDE (2013)	46	33	58	109
Soups and broths	Group I	QS	Intertek Scientific & Regulatory Consultancy (2014)	560	560	560	1050
Sauces	Group I	QS	FDE (2007)	5000	5000	5000	5000
Sauces	Group I	QS	FDE (2013)	1038	54	2078	9900
Sauces	Group I	QS	FDE (2014)	3000	3000	3000	3300
Sauces	Group I	QS	Intertek Scientific & Regulatory Consultancy (2014)	1460	1400	2000	3500
Sauces	Group I	QS	Intertek Scientific & Regulatory Consultancy (2014)	2667	2000	3000	5000
Salads and savoury-based sandwich spreads	Group I	QS	Intertek Scientific & Regulatory Consultancy (2014)	1400	1400	1400	3500
Protein products, excluding products covered in category 1.8	Group I	QS	Intertek Scientific & Regulatory Consultancy (2014)	2627	480	5000	8000
Protein products, excluding products covered in category 1.8	Group I	QS	Intertek Scientific & Regulatory Consultancy (2014)	3000	3000	3000	5000
Follow-on formulae as defined by Directive 2006/141/EC	E 407	300mg/Kg	Intertek Scientific & Regulatory Consultancy (2014)	300	300	300	300
Other foods for young children	E 407	300mg/Kg	Intertek Scientific & Regulatory Consultancy (2014)	70	70	70	210
Other foods for young children	E 407	300mg/Kg	SNE (2014)	110	110	110	110
Dietary foods for infants for special medical purposes and special formulae for infants	E 407	300mg/Kg	SNE (2014)	300	300	300	300
Dietary foods for babies and young children for special medical purposes as defined in Directive 1999/21/EC	E 407	300mg/Kg	SNE (2014)	300	300	300	300
Dietary foods for special medical purposes defined in Directive 1999/21/EC (excluding products from food category 13.1.5)	Group I	QS	SNE (2014)	920	80	8000	8000
Dietary foods for weight control diets intended to replace total daily food intake or an individual meal (the whole or part of the total daily diet)	Group I	QS	FDE (2013)	177	177	177	177
Dietary foods for weight control diets intended to replace total daily food intake or an individual meal (the whole or part of the total daily diet)	Group I	QS	Intertek Scientific & Regulatory Consultancy (2014)	1700	1400	2000	3000
Dietary foods for weight control diets intended to replace total daily food intake or an individual meal (the whole or part of the total daily diet)	Group I	QS	Intertek Scientific & Regulatory Consultancy (2014)	1000	1000	1000	10000
Dietary foods for weight control diets intended to replace total daily food intake or an individual meal (the whole or part of the total daily diet)	Group I	QS	SNE (2014)	4200	4200	4200	5000
Dietary foods for weight control diets intended to replace total daily food intake or an individual meal (the whole or part of the total daily diet)	Group I	QS	SNE (2014)	455	455	455	455
Flavoured drinks	Group I	QS	FDE (2013)	107	107	107	107
Flavoured drinks	Group I	QS	FDE (2014)	800	251	1800	14811
Flavoured drinks	Group I	QS	FDE (2014)	100	100	100	450
Flavoured drinks	Group I	QS	Intertek Scientific & Regulatory Consultancy (2014)	260	150	1000	2000
Flavoured drinks	Group I	QS	Intertek Scientific & Regulatory Consultancy (2014)	192	150	200	500
Flavoured drinks	Group I	QS	Rudolf Wild GmbH & Co. KG (2014)	180	180	180	300

Other	Group I	QS	FDE (2014)	65	65	65	250
			Intertek Scientific & Regulatory Consultancy (2014)	200	200	200	500
Spirit drinks as defined in Regulation (EC) No 110/2008	Group I	QS	Intertek Scientific & Regulatory Consultancy (2014)	490	490	490	700
Aromatised wine-based drinks	Group I	QS	Intertek Scientific & Regulatory Consultancy (2014)	1000	1000	1000	2000
Other alcoholic drinks including mixtures of alcoholic drinks with non-alcoholic drinks and spirits with less than 15 % of alcohol	Group I	QS	FDE (2014)	20	20	20	20
			Intertek Scientific & Regulatory Consultancy (2014)	570	140	1000	2000
Desserts excluding products covered in category 1, 3 and 4	Group I	QS	Delixia s.r.l. (2014)	1666	1666	1666	2000
			EUROGUM A/S (2014)	5000	5000	5000	20000
			FDE (2013)	2010	190	5357	8476
			FDE (2014)	321	74	460	460
			FDE (2014)	1768	240	4100	4200
			Intertek Scientific & Regulatory Consultancy (2014)	2737	150	6000	12000
			Rudolf Wild GmbH & Co. KG (2014)	780	450	1110	3000
Food supplements supplied in a solid form including capsules and tablets and similar forms, excluding chewable forms	Group I	QS	AESPG (2014)	50488	50488	50488	50488
			Intertek Scientific & Regulatory Consultancy (2014)	25857	140	40000	60000
			Intertek Scientific & Regulatory Consultancy (2014)	35000	35000	35000	50000
Food supplements supplied in a liquid form	Group I	QS	Intertek Scientific & Regulatory Consultancy (2014)	1542	1500	2000	3000
Food supplements supplied in a syrup-type or chewable form	Group I	QS	Intertek Scientific & Regulatory Consultancy (2014)	5000	5000	5000	8000

E 407: carrageenan; E 407a: processed Eucheuma seaweed; E 407 and E 407a are included in the Group I of food additives authorised at quantum satis (QS).
MPL: Maximum permissible level
FDE: Food Drink Europe
SNE: Specialised Nutrition Europe

1. Additives EPoF, Food NSat, Younes M, et al. Re-evaluation of carrageenan (E 407) and processed Eucheuma seaweed (E 407a) as food additives. *EFSA journal.* 2018;16(4):e05238.

Supplementary figure legends

Figure S1. Carrageenan-induced apoptosis detected using FITC annexin V/PI double staining and flow cytometric analysis. Q1: early apoptotic cells; Q2: late apoptotic cells; Q3:dead cells; Q4: live cells. κ-CGN: κ-carrageenan; ι-CGN: ι-carrageenan; λ-CGN: λ-carrageenan.

Figure S2. Heatmap of the differentially expressed genes. Genes involved in IL-17 signaling pathway, cellular response to chemokine, NF-kappa B signaling pathway, and cholesterol metabolism that were significantly altered in response to κ-, ι-, λ-carrageenan at the concentration of 50 and 5000 μg/mL vs unexposed control. Each column represents a sample, and each row represents a gene. The red and blue colors indicate gene upregulation and downregulation, respectively. κ-CGN: κ-carrageenan; ι-CGN: ι-carrageenan; λ-CGN: λ-carrageenan.

Figure S3. Violin plots showing differentially expressed genes in response to κ-, ι-, λ-carrageenan at the concentrations of 50 and 5000 μg/mL. *$p < 0.05$, ** $p < 0.01$, *** $p < 0.001$. NGX, normalized gene expression. κ-CGN: κ-carrageenan; ι-CGN: ι-carrageenan; λ-CGN: λ-carrageenan.

Figure S4. Volcano plots showing differentially expressed genes in response to κ-, ι-, λ-carrageenan at the concentration of 5000 μg/mL after 24 hours. Upregulated genes are marked in red and downregulated genes are shown in blue. The x-axis showed log2(fold change) in expression and the y-axis log10(p value) of differentially expressed genes. κ-CGN: κ-carrageenan; ι-CGN: ι-carrageenan; λ-CGN: λ-carrageenan.

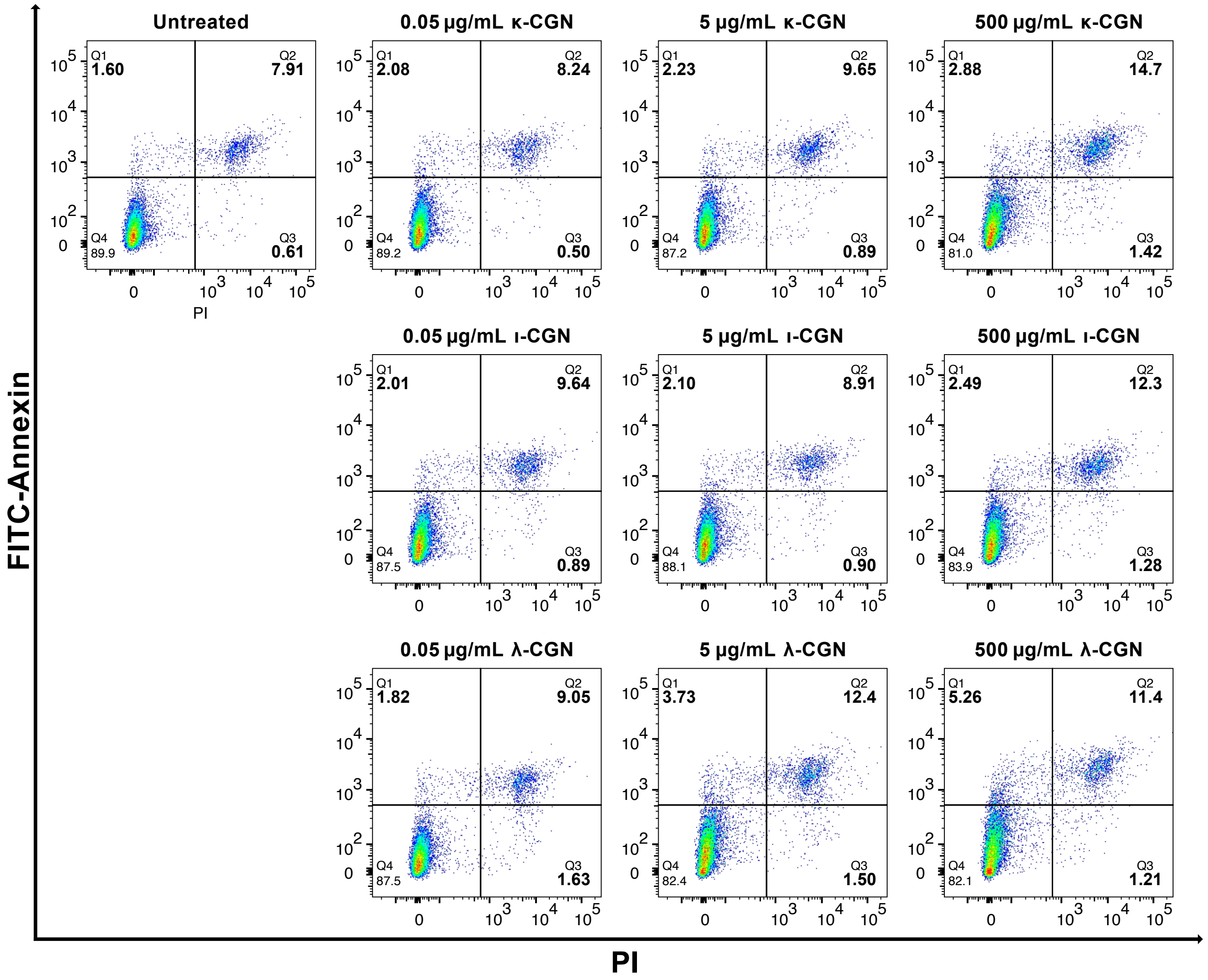

Supplementary Figure 1

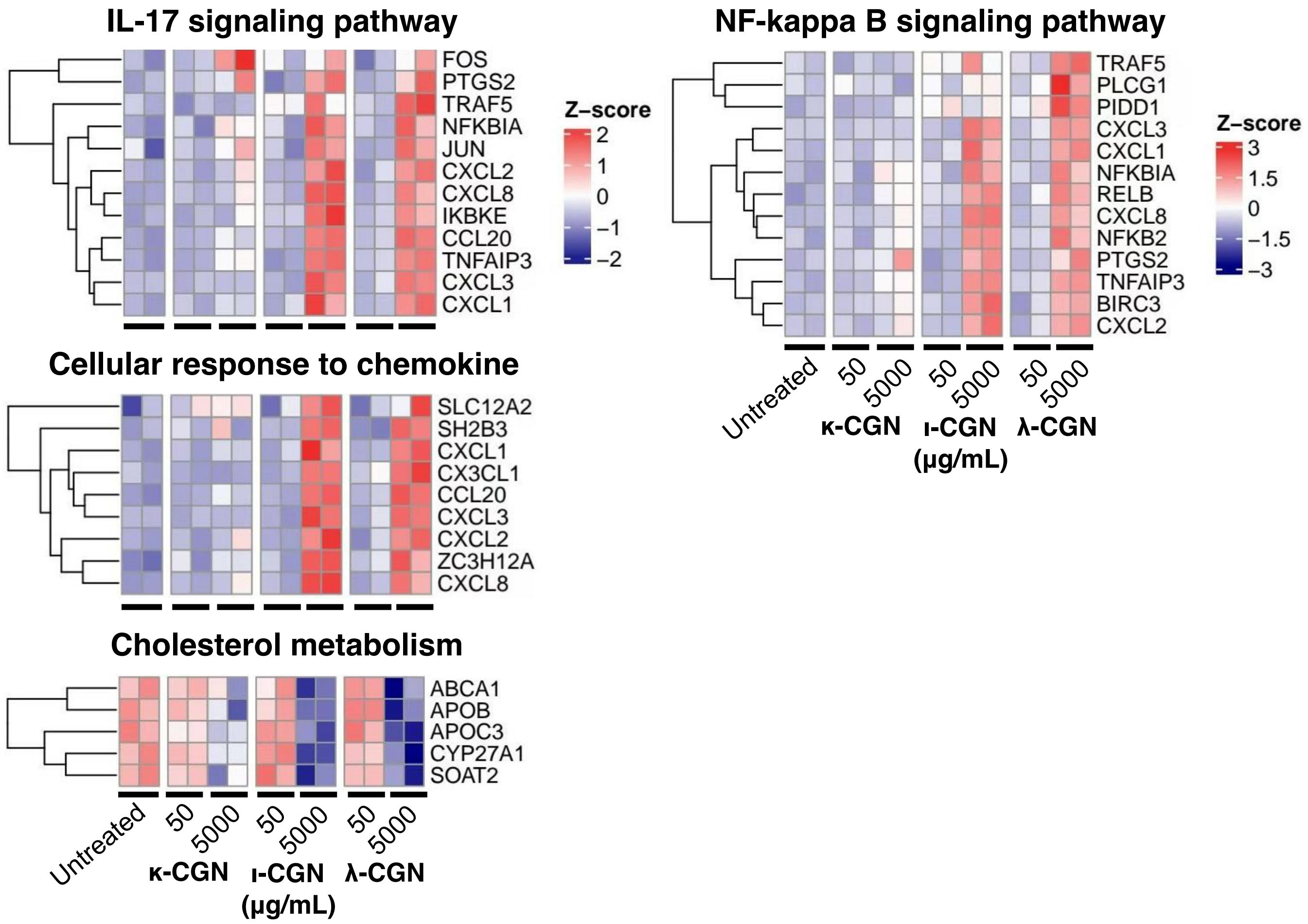

Supplementary Figure 2

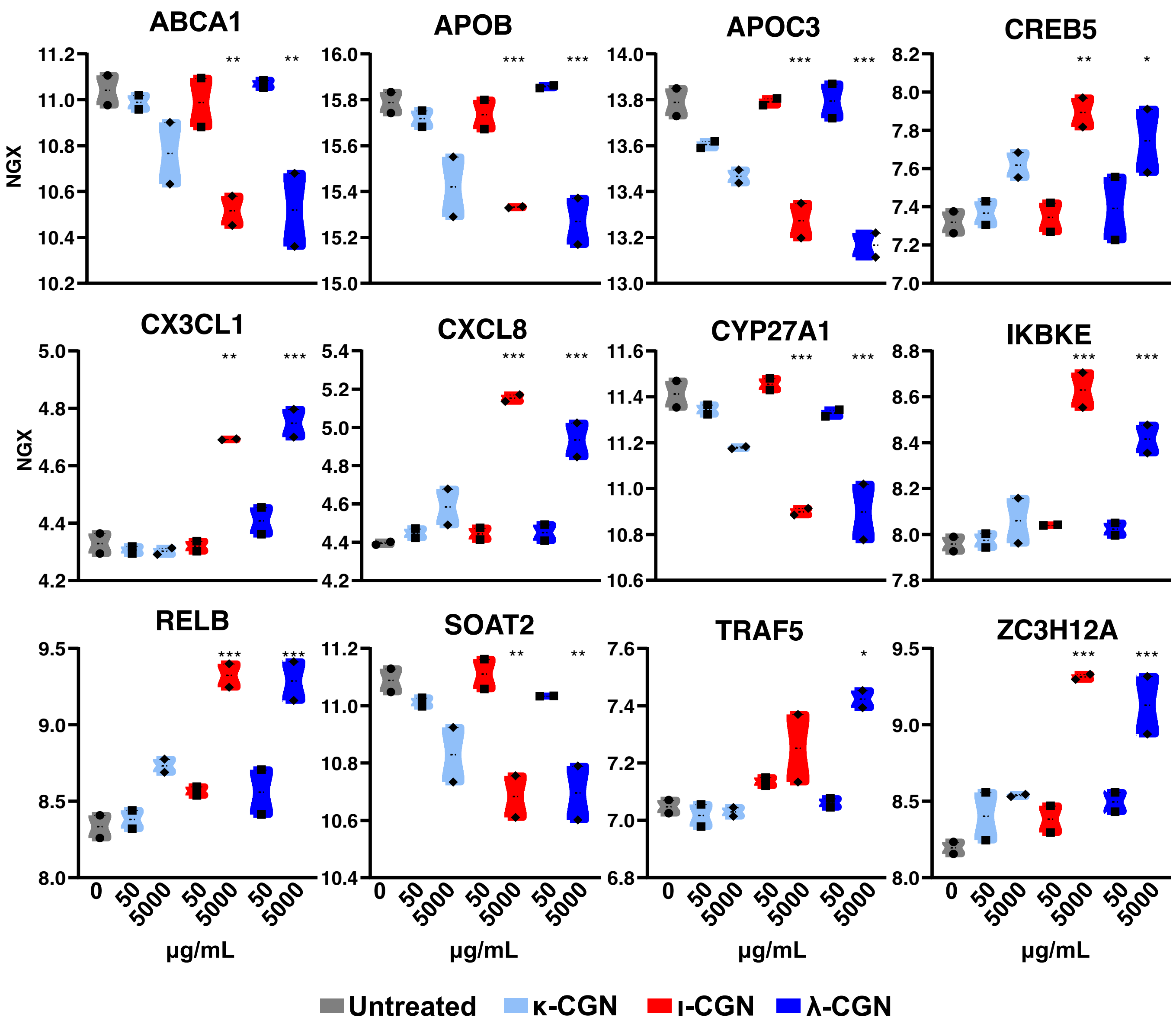

Supplementary Figure 3

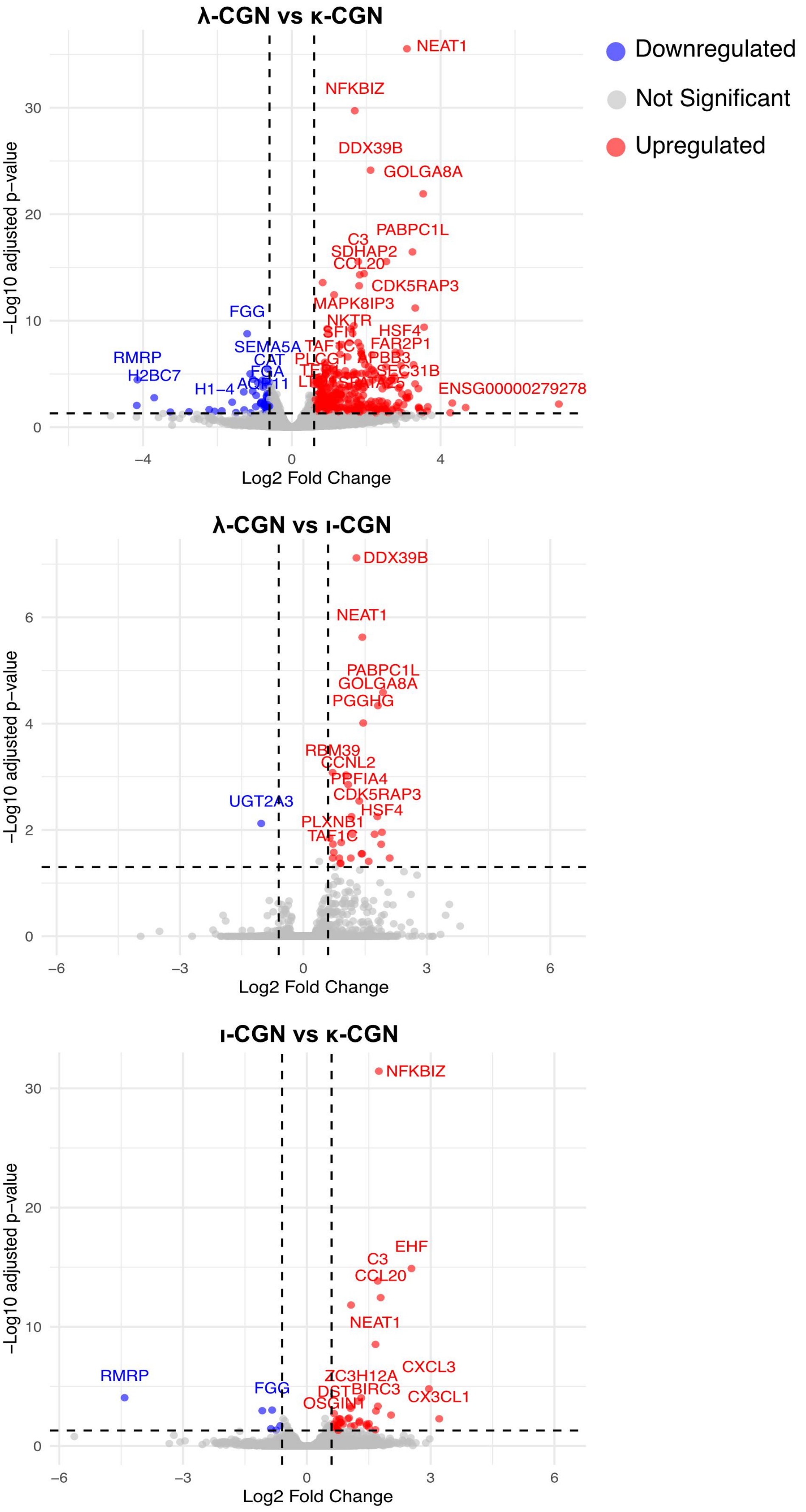

Supplementary Figure 4
